# Oxidative balance score and menopausal status: insights from epidemiological analysis and machine learning models

**DOI:** 10.3389/fnut.2025.1586606

**Published:** 2025-05-20

**Authors:** Chunlin Dong, Ding Ma, Jinjin Yu, Ke Gu, Yaying Lin, Jing Song, Yuan Wang, Yanjun Zhou

**Affiliations:** ^1^Department of Obstetrics and Gynecology, Affiliated Hospital of Jiangnan University, Wuxi, China; ^2^Wuxi Medical College, Jiangnan University, Wuxi, China; ^3^Key Laboratory of the Ministry of Education, Cancer Biology Research Center, Tongji Hospital, Tongji Medical College, Huazhong University of Science and Technology, Wuhan, China; ^4^Department of Radiation Oncology, Affiliated Hospital of Jiangnan University, Wuxi, China

**Keywords:** menopause, oxidative stress, oxidative balance score, niacin, magnesium

## Abstract

**Background:**

Unhealthy lifestyle habits, such as smoking, can impact oxidative stress. During oxidative stress, unnaturalized free radicals can damage DNA, proteins, and lipids, leading to cellular damage and death. A comprehensive measurement of various pro-oxidative and antioxidative exposures can reflect an individual's oxidative stress burden. However, studies on assessing the association between dietary and lifestyle factors related to oxidative stress and menopause were previously lacking.

**Materials and methods:**

A cohort of 2,813 women aged 40–60 years from the National Health and Nutrition Examination Survey conducted between 2003 and 2020 was identified as meeting the eligibility criteria. The associations of oxidative balance score (OBS) with the menopausal status were examined via weighted logistic regression models, and the odds ratios (ORs) of menopause onset were calculated with 95% confidence intervals (CIs). Machine learning models were developed and compared to classify the menopausal status based on the OBS and other epidemiological factors, with the interpretability of the models explored using the Shapley Additive Explanations method.

**Results:**

Following adjustment for various confounding factors, OBS was reversely associated with menopause (OR: 0.97, 95% CI: 0.94–0.99, *p* = 0.010). When the OBS was categorized into quartiles, the association with menopause was still significant (*p* for trend = 0.009). The association of the OBS with menopause remained significant after excluding any each survey year cycles (*p* for trend < 0.050). The menopause classification models developed using TabFPN, Random Forest, CatBoost, and XGBoost achieved an area under the curve of 0.880, 0.884, 0.886, and 0.878, respectively. Based on the results from epidemiological analysis and machine learning models, the intake of magnesium, zinc, niacin, and vitamin B6 showed a decline in the early postmenopausal period and contributed in the model performance.

**Conclusions:**

OBS were reversely associated with the menopausal status, and the OBS might serve as an indicator of an individual's oxidative stress status for lifestyle interventions during the menopausal transition.

## 1 Introduction

Menopause refers to the permanent cessation of menstruation, which signifies the depletion of oocytes in ovarian follicles. Menopause is directly associated with ovarian aging (1). Females are born with a predetermined number of ovarian follicles. The impact of detrimental dietary and lifestyle behaviors on oocyte aging and death, potentially influencing the likelihood of menopause, lacks consensus among researchers.

Oxidative stress (OS) is defined as a state of dysregulation between free radicals and the antioxidant systems of the cells (2). Free radicals encompass reactive oxygen species (ROS), reactive nitrogen species (RNS), and other reactive species with unpaired electrons (3). ROS refer to the products obtained from the two-electron reduction of oxygen, including superoxide anions, hydrogen peroxide, hydroxyl radicals, lipid peroxides, protein peroxides, and peroxides derived from nucleic acids. They play a crucial role in maintaining a dynamic balance in biological systems through a series of redox reactions and act as signaling molecules that regulate cellular pathways. In a state of normal physiological functioning, cells employ antioxidant defense mechanisms to counterbalance oxidative stress and uphold redox equilibrium. Nevertheless, if the generation of oxidants surpasses the organism's antioxidant capacity, oxidative stress arises. The accumulation of oxidants within cells instigates a series of harmful reactions that compromise the integrity of proteins, lipids, and DNA, ultimately leading to cellular dysfunction and potential cell death (4, 5). Therefore, the excessive accumulation of ROS ultimately leads to cellular dysfunction and apoptosis (5). Unhealthy lifestyle habits, such as smoking, contribute to an increased generation of ROS and are associated with early natural menopause (6, 7). The existing literature is insufficient in exploring the association of menopause with the intake of antioxidants and unhealthy lifestyle habits.

In order to assess the OS caused by lifestyle and dietary patterns, various algorithms for oxidative balance score (OBS) have been developed (8). The OBS utilized in this study consists of two components: dietary OBS and lifestyle OBS, which is significantly associated with the telomere length, a biomarker of cellular aging (9). The dietary OBS encompasses the intake of dietary fiber, carotene, riboflavin, niacin, vitamin B6, total folate, vitamin B12, vitamin C, vitamin E, calcium, magnesium, zinc, copper, selenium, total fat, and iron. Lifestyle OBS components include physical activity, alcohol consumption, body mass index (BMI), and serum cotinine levels. OBS has been reported to be associated with phenotypic age acceleration and osteoporosis (10). Currently, there is no existing research on the association between OBS and menopause. Therefore, this study utilized the NHANES database from 2003 to 2020 to investigate the association between OBS and the odds of menopause in women aged 40–60 years.

A variety of machine learning models are employed in classification tasks, with each algorithm presenting distinct strengths and capabilities. The tabular prior-data fitted network (TabPFN), which is a tabular foundation model, demonstrates a marked superiority over all prior methods across various datasets containing up to 10,000 samples, accompanied by a significant reduction in training time (11). Categorical boosting (CatBoost), extreme gradient boosting (XGBoost) and light gradient boosting machine, are three algorithms referred to as gradient boosting decision tree (GBDT), all of which represent improved implementations within the GBDT framework. CatBoost is a GBDT framework that utilizes symmetric decision trees (oblivious trees) as base learners, characterized by a lower number of parameters, support for categorical variables, and high accuracy. To identify high-odds menopausal women, four machine learning algorithms were employed to establish menopause classification models for women aged 40 to 60, based on epidemiological information and various components of the OBS. The models were then compared on a testing set. Additionally, SHapley Additive exPlanations (SHAP) were integrated to elucidate the interpretability of the menopause classification models. This interpretability is expected to facilitate broader acceptance of these established models in real-world clinical settings.

Menopause has significant implications for clinical practice and public health. Women who experience menopause at a later age are likely to have higher levels of hormones and longer durations of estrogen exposure, which may be associated with an elevated risk of endometrial and breast carcinoma (12). However, early menopause is associated with an increased risk of cardiovascular disease due to the premature loss of estrogen's protective effects on the cardiovascular system (13). Therefore, the primary objective of this study was to provide exploratory lifestyle and dietary intervention strategies for premenopausal women through epidemiological analysis and machine learning models, and to conduct a preliminary exploration of the association between dietary and lifestyle-induced OS and menopause. Identifying intervention-sensitive periods and implementing early intervention strategies, such as adopting a low OS lifestyle and dietary habits, represent crucial opportunities to mitigate the odds of cardiovascular disease and cancer development.

## 2 Methods

### 2.1 Study design

The NHANES is a cross-sectional survey in the United States that gathers health and nutritional information representative of the entire population. The National Center for Health Statistics Ethics Review Board provided ethical approval for the protocols of the NHANES surveys. The details of the approved protocols for each survey cycle can be found in Supplementary Table 1. All participants provided written consent after being informed about the details of the study. The data used in this analysis are publicly accessible and do not include any personally identifiable information. The analytical procedure was performed in accordance with the applicable guidelines and regulations.

Information on participants' age, race, educational attainment, partnership, family income, tobacco usage behavior, alcohol consumption, and metabolic equivalent (MET) of physical activity (PA) was collected at participants' homes. Subsequently, a standardized physical examination, anthropometric assessments, blood sample collection, a 24-h dietary recall interview, and additional inquiries were conducted at a mobile examination center. The 24-h dietary recall interviews involved participants detailing the types and amounts of foods and beverages they consumed in the preceding 24 hours. This information was subsequently captured in NHANES's computer-assisted dietary interview system. The evaluation of dietary nutrient intake relied on the Food Intake Analysis System from the University of Texas and the Nutrient Database provided by the U.S. Department of Agriculture. It is important to note that these nutrient estimates did not include any contributions from dietary supplements or medications. The weekly MET values were computed based on individual-specific leisure activity data from the past 30 days, gathered through household interviews.

### 2.2 Menopause assessment

Participants answered the question: “Have you had at least one menstrual period in the past 12 months? (This excludes any vaginal bleeding resulting from medical conditions, hormone therapy, or surgical interventions).” If the answer was NO, the participants need to respond to the next question, “What is the reason that you have not had a period in the past 12 months?” After excluding those who were pregnant or breastfeeding, individuals who have not menstruated for 12 months were considered to be menopausal. A total of 2,813 female participants aged 40–60 years with documented menstrual statuses from 2003 to 2020 were included in this study. Postmenopausal years were calculated by subtracting the self-reported age at menopause from the age at the time of the survey.

### 2.3 OBS

The calculation formula for the OBS was described in Zhang et al. (9). The OBS for female participants was calculated by assigning scores (0, 1, or 2) to 20 components. Dietary OBS components include: dietary fiber: 0 (< 10.10 g/d), 1 (10.10–16.31 g/d), 2 (≥16.31 g/d); carotene (retinol equivalents): 0 (< 98.08 RE/d), 1 (98.08–383.50 RE/d), 2 (≥383.50 RE/d); riboflavin: 0 (< 1.34 mg/d), 1 (1.34–2.02 mg/d), 2 (≥2.02 mg/d); niacin: 0 (< 14.52 mg/d), 1 (14.52–21.86 mg/d), 2 (≥21.86 mg/d); vitamin B6: 0 (< 1.13 mg/d), 1 (1.13–1.77 mg/d), 2 (≥1.77 mg/d); total folate: 0 (< 251.00 μg/d), 1 (251.00–388.96 μg/d), 2 (≥388.96 μg/d); vitamin B12: 0 (< 2.22 μg/d), 1 (2.22–4.22 μg/d), 2 (≥4.22 μg/d); vitamin C: 0 (< 38.01 mg/d), 1 (38.01–98.49 mg/d), 2 (≥98.49 mg/d); vitamin E (α-tocopherol equivalents): 0 (< 4.53 mg/d), 1 (4.53–7.52 mg/d), 2 (≥7.52 mg/d); calcium: 0 (< 499.24 mg/d), 1 (499.24–849.00 mg/d), 2 (≥849.00 mg/d); magnesium: 0 (< 187.00 mg/d), 1 (187.00–283.43 mg/d), 2 (≥283.43 mg/d); zinc: 0 (< 6.73 mg/d), 1 (6.73–10.75 mg/d), 2 (≥10.75 mg/d); copper: 0 (< 0.85 mg/d), 1 (0.85–1.28 mg/d), 2 (≥1.28 mg/d); selenium: 0 (< 67.79 μg/d), 1 (67.79–99.50 μg/d), 2 (≥99.50 μg/d); total fat: 0 (≥75.79 g/d), 1 (50.98–75.79 g/d), 2 (< 50.98 g/d); iron: 0 (≥14.32 mg/d), 1 (9.65–14.32 mg/d), 2 (< 9.65 mg/d). Lifestyle OBS components include: physical activity: 0 (< 270.00 MET-min/week), 1 (270.00–845.71 MET-min/week), 2 (≥845.71 MET-min/week); alcohol consumption: 0 (≥15 g/d), 1 (0–15 g/d), 2 (none); BMI: 0 (≥28.64 kg/m^2^), 1 (23.74–28.64 kg/m^2^), 2 (< 23.74 kg/m^2^); serum cotinine levels: 0 (≥0.172 ng/mL), 1 (0.035–0.172 ng/mL), 2 (< 0.035 ng/mL); physical activity: 0 < 270, 1 = 270–845.71, 2 ≥ 845.71 MET-min/week. In the calculation of OBS, serum cotinine levels were employed as a biomarker for tobacco use. Cotinine, which is the principal metabolite of nicotine and has a longer half-life, is used to quantify tobacco consumption and evaluate exposure to environmental tobacco smoke. The measurements were conducted using isotope dilution high-performance liquid chromatography (HPLC) combined with atmospheric pressure chemical ionization tandem mass spectrometry (APCI-MS/MS), specifically employing a Hewlett-Packard model 1090L for HPLC and a PE-Sciex API III triple quadrupole mass spectrometer for APCI-MS/MS analysis. Among the components, pro-oxidant factors were reverse scored, including total fat intake, iron intake, alcohol consumption, serum cotinine levels (smoking exposure biomarker), and BMI, while the remaining components were considered antioxidants and positively scored. The total OBS (range 0–34) was derived by summing all component scores, with higher values indicating stronger antioxidant/weaker pro-oxidant profiles.

### 2.4 Covariates

Regarding the selection of covariates, in the original text proposing the OBS calculation method, the following variables were adjusted: age, race, education, and Poverty Income Ratio (PIR) (9). These indicators are known to influence individuals' lifestyles and dietary habits; consequently, they were incorporated as covariates in this study. Additionally, daily energy intake was identified as a direct determinant of the intake levels of OBS components. Participants with hypertension, CVD, DM, or hyperlipidemia may have lower intakes of vegetables, fruits, whole grains, low-fat dairy products, and seafood, alongside higher consumptions of red and processed meats, refined grains, and sugar-sweetened foods and beverages (14). However, participants with hypertension, CVD, DM, or hyperlipidemia often adhere to specific dietary regimens, such as low-sodium and low-fat diets, low glycemic index foods, and controlled carbohydrate intake, which can influence the OBS calculation. Therefore, hypertension, CVD, DM, and hyperlipidemia were included as covariates in the model. Lifestyle factors such as smoking and alcohol consumption directly affect the body's redox system, with smoking leading to an increase in ROS (6, 7). Smoking and alcohol consumption can influence the functional pathways of vitamin B6 in the body (15). The menopausal transition, a decline in estrogen levels frequently results in weight gain and metabolic disturbances in women, thereby influencing the body's oxidative balance (16, 17). Thus, BMI and fasting blood glucose were included as covariates. Furthermore, individuals with partners tend to adhere more closely to a healthy lifestyle, characterized by regular dietary and exercise habits, which contribute to the maintenance of oxidative balance.

The diagnostic criteria for hypertension, hyperlipidemia, cardiovascular disease (CVD), and diabetes mellitus were previously described (18). Briefly, hyperlipidemia was diagnosed with triglycerides ≥150 mg/dL, total cholesterol ≥200 mg/dL, LDL ≥130 mg/dL, HDL < 40 mg/dL, or the use of antihyperlipidemic therapy. Cardiovascular disease (CVD) was recognized through previous myocardial infarction or cerebrovascular events. Type 2 diabetes mellitus (DM) was diagnosed according to any of the following criteria: prior diabetes diagnosis, HbA1c ≥ 6.5%, fasting blood glucose ≥7.0 mmol/L, random blood glucose ≥11.1 mmol/L, oral glucose tolerance test ≥11.1 mmol/L, or the use of antihyperglycemic medications. Hypertension was identified if participants had a history of hypertension, were prescribed antihypertensive drugs, or had systolic blood pressure ≥140 mmHg or diastolic blood pressure ≥90 mmHg.

The procedures for blood testing are comprehensively outlined and readily available (19). Briefly, fasting serum biochemistry profiles, including aspartate aminotransferase (AST), alanine aminotransferase (ALT), gamma-glutamyl transferase (GGT), uric acid, low-density lipoprotein (LDL), glucose, glycated hemoglobin (HbA1c), blood urea nitrogen, albumin, and total bilirubin, were measured using a Hitachi 704 Analyzer (Roche/Boehringer Mannheim Corporation, Indianapolis, USA). The weight “wtasf2yr” was employed as a weighting factor in the weighted analysis.

### 2.5 Statistical analyses

All statistical analyses were implemented using R programming language. Continuous variables related to the characteristics of the females were documented in the form of means (standard errors) or means (95% CIs), and comparisons were performed via one-way ANOVA. Categorical factors were recorded as frequency (percentage) and assessed via the chi-square test. Weighted restricted cubic splines (RCSs) from the “rms” package were utilized to examine non-linear relationships. The associations between menopause odds and the OBS were analyzed using weighted logistic regression models to calculate odds ratios (ORs) along with their associated 95% confidence intervals (CIs) utilizing the R package “survey”. The crude model did not incorporate any adjustments. Model 1 based on the crude model was built with the adjustment for age, race, PIR, education, single status, BMI, cigarette consumption, alcohol intake. Model 2 was built upon Model 1 by further incorporating adjustments for medical history variables, including hypertension, CVD, DM, and hyperlipidemia, along with fasting glucose, and daily energy intake. To ensure the robustness of the model results and to avoid overfitting due to limited sample sizes, the sensitivity analyses were conducted. The weighted multivariable logistic regression was conducted with the survey years sequentially excluded in the Model 2. The Weighted ROC package was used to calculate the AUC under the condition of sequentially excluding survey year cycles. A difference in AUC (ΔAUC) >0.15 indicates a risk of overfitting across different datasets. Weighted stratified analysis was conducted based on Model 2, excluding the stratification factors from the covariates. Additionally, the *p* for interaction was adjusted using the Benjamini-Hochberg (BH) method to account for false positives due to multiple testing. Linear regression analyses were conducted to examine the associations between the duration of time since menopause and OBS, as well as between age and OBS, with the covariates in the Model 2. Significance level was set at *p* < 0.05.

### 2.6 Establishment of classification models

The models were developed using the sklearn library and the tabpfn library in Python 3.8. The cohort was randomly divided into 50% for the training set, while the remaining 50% was reserved for the validation set. Within the training set, the five-fold cross-validation was employed to guarantee the model's generalizability and assessed the AUC values of four machine learning models. For assessing model discrimination ability, the area under the receiver operating characteristic curve (AUROC), the area under the precision-recall curve (AUPRC), sensitivity, specificity, and accuracy were computed in the testing set. In elucidating the contribution of each feature to the “black-box” classifications, SHAP is utilized to analyze the classfications made by the classifier. The Shapley values quantify the marginal impact of each feature on the model's final prediction.

## 3 Results

### 3.1 Characteristics of female participants

The participants were divided into premenopausal group and postmenopausal group. The indicators, including age (in years), alkaline phosphatase, albumin, gamma-glutamyl transferase, uric acid, blood urea nitrogen, iron, fasting triglycerides, fasting total cholesterol, LDL cholesterol, fasting glucose, MET of total physical activity, HbA1c, ALT, AST, the percentage of singles, as well as the incidence rates of hyperlipidemia, CVD, DM, and hypertension, were observed to be elevated in postmenopausal women compared to premenopausal women (Table 1, *p* < 0.050). The composition of race, years of education, and smoking status differed significantly between the two groups (Table 1, *p* < 0.050).

**Table 1:** Baseline characteristics of premenopausal and postmenopausal women.

**Variables**	**Pre- menopause**	**Post- menopause**	***p*-value**
Age	45.81(0.15)	53.16(0.17)	< 0.001
PIR	3.30(0.07)	3.25(0.06)	0.610
Energy intake (kcal/day)	1,890.22(25.71)	1,735.38(18.38)	< 0.001
Fast glucose (mg/dl)	100.51(0.70)	107.14(1.03)	< 0.001
HbA1c (%)	5.51(0.02)	5.74(0.03)	< 0.001
ALT (U/L)	20.17(0.45)	23.33(0.42)	< 0.001
AST (U/L)	22.17(0.48)	24.37(0.53)	0.002
Bilirubin total (umol/L)	10.58(0.16)	10.32(0.15)	0.248
Alkaline phosphatase (u/L)	63.06(0.67)	75.53(0.82)	< 0.001
Albumin (g/L)	40.75(0.12)	41.60(0.11)	< 0.001
Gamma glutamyl transferase (U/L)	21.33(0.57)	29.34(1.13)	< 0.001
Creatinine (mg/dl)	0.75(0.01)	0.77(0.01)	0.049
Uric acid (umol/L)	274.34(2.36)	295.71(2.24)	< 0.001
Blood urea nitrogen (mmol/L)	4.00(0.05)	4.71(0.05)	< 0.001
Iron (umol/L)	14.11(0.23)	15.61(0.21)	< 0.001
Fast triglyceride (mmol/L)	1.27(0.04)	1.48(0.04)	< 0.001
Fast total cholesterol (mmol/L)	5.09(0.04)	5.48(0.04)	< 0.001
HDL cholesterol (mmol/L)	1.56(0.02)	1.55(0.02)	0.786
LDL cholesterol (mmol/L)	2.97(0.03)	3.26(0.03)	< 0.001
Race			0.022
Non-Hispanic white	507(68.85)	673(71.61)	
Non-Hispanic black	271(11.59)	393(12.20)	
Mexican American	206(7.78)	217(5.12)	
Other	247(11.78)	299(11.08)	
Education			0.004
< 9 years	72(3.34)	108(3.27)	
9–12 years	385(26.89)	557(33.89)	
>12 years	774(69.77)	917(62.84)	
Single status			0.041
Single	421(29.71)	664(34.43)	
Not single	810(70.29)	918(65.57)	
Smoking			0.001
Never	803(63.17)	870(53.32)	
Former	207(19.18)	358(24.34)	
Now	221(17.65)	354(22.34)	
Alcohol uptake			0.192
Never	180(10.73)	263(12.80)	
Former	147(11.73)	245(13.28)	
Now	904(77.53)	1,074(73.92)	
Hyperlipidemia			< 0.001
No	416(33.12)	266(16.17)	
Yes	815(66.88)	1,316(83.83)	
CVD			< 0.001
No	1,179(96.74)	1,423(91.85)	
Yes	52(3.26)	159(8.15)	
DM			< 0.001
No	1,059(89.49)	1,215(81.75)	
Yes	172(10.51)	367(18.25)	
Hypertension			< 0.001
No	852(71.08)	757(53.57)	
Yes	379(28.92)	825(46.43)	

Continuous variables are presented as mean (standard error) and categorical variables are presented frequency (percentage). OBS, Oxidative Balance Score; PIR, poverty income ratio; BMI, body mass index; HbA1c, glycated hemoglobin A1c; ALT, Alanine Aminotransferase; AST, Aspartate Aminotransferase; HDL, high-density lipoprotein; LDL, low-density lipoprotein; CVD, cardiovascular disease; DM, diabetes mellitus.

Then, dietary intake and lifestyle patterns were compared between premenopausal and postmenopausal women based on 24-h dietary recalls, comprehensive questionnaires, and anthropometric measurements (Table 2). Postmenopausal women revealed distinct dietary and lifestyle patterns. Postmenopausal women exhibited significantly lower intake of dietary fiber (14.83 ± 0.26 vs. 15.78 ± 0.30 g/day, *p* = 0.01), total fat (68.27 ± 0.93 vs. 73.70 ± 1.27 g/day, *p* < 0.001), and several micronutrients, including niacin (20.44 ± 0.26 vs. 22.22 ± 0.32 mg/day, *p* < 0.001), vitamin B6 (1.65 ± 0.02 vs. 1.78 ± 0.03 mg/day, *p* < 0.001), total folate (338.70 ± 5.07 vs. 373.24 ± 7.06 mcg/day, *p* < 0.0001), riboflavin (1.88 ± 0.02 vs. 2.00 ± 0.03 mg/day, *p* < 0.001), and minerals such as magnesium (262.57 ± 3.42 mg vs. 282.12 ± 4.73 mg, *p* < 0.001), calcium, iron, zinc, copper, and selenium (*p* < 0.01). Conversely, no significant differences were observed in alpha-carotene, beta-carotene, vitamin B12, vitamin C, vitamin E, or BMI (*p* > 0.050). PA was higher in postmenopausal women (2819.10 ± 180.48 vs. 2397.36 ± 122.79 MET, *p* = 0.040), while alcohol consumption was lower (5.47 ± 0.44 vs. 7.38 ± 0.72 g/day, *p* = 0.020).

**Table 2:** Comparative analysis of OBS and the components between premenopausal and postmenopausal women.

**Variable**	**Pre- menopause**	**Post- menopause**	***p*-value**
Dietary fiber (g)	15.78(0.30)	14.83(0.26)	0.012
Total fat (g)	73.70(1.27)	68.27(0.93)	< 0.001
Alpha carotene (mcg)	411.15(24.17)	384.73(21.00)	0.388
Beta carotene (mcg)	2,284.86(95.68)	2,371.48(91.25)	0.499
Riboflavin (mg)	2.00(0.03)	1.88(0.02)	< 0.001
Niacin (mg)	22.22(0.32)	20.44(0.26)	< 0.001
Vitamin B6 (mg)	1.78(0.03)	1.65(0.02)	< 0.001
Total folate (mcg)	373.24(7.06)	338.70(5.07)	< 0.001
Vitamin B12 (mcg)	4.27(0.10)	4.03(0.11)	0.098
Vitamin C (mg)	76.03(2.41)	71.72(1.98)	0.129
Vitamin E ATE (mg)	7.95(0.19)	7.50(0.16)	0.069
Calcium (mg)	880.56(16.70)	817.45(13.16)	0.002
Magnesium (mg)	282.12(4.73)	262.57(3.42)	< 0.001
Iron (mg)	13.64(0.26)	12.38(0.16)	< 0.001
Zinc (mg)	10.27(0.17)	9.32(0.13)	< 0.001
Copper (mg)	1.19(0.02)	1.12(0.02)	0.004
Selenium (mcg)	101.06(1.54)	93.14(1.35)	< 0.001
Alcohol (g)	7.38(0.72)	5.47(0.44)	0.020
BMI (kg/m^2^)	29.75(0.30)	29.98(0.28)	0.545
Total PA (MET)	2,397.36(122.79)	2,819.10(180.48)	0.040
Cotinine (ng/ml)	48.74(4.96)	58.11(4.69)	0.088
Carotene (RE)	207.54(8.74)	213.65(8.18)	0.598
Score dietary fiber (g)	1.16(0.03)	1.06(0.03)	0.010
Score carotene (RE)	0.70(0.02)	0.72(0.02)	0.532
Score riboflavin (mg)	1.23(0.03)	1.13(0.03)	0.015
Score niacin (mg)	1.29(0.03)	1.15(0.03)	< 0.001
Score vitamin B6 (mg)	1.25(0.03)	1.11(0.03)	< 0.001
Score total folate (mcg)	1.11(0.03)	0.97(0.03)	< 0.001
Score vitamin B12 (mcg)	1.23(0.03)	1.12(0.03)	0.007
Score vitamin C (mg)	0.94(0.03)	0.90(0.03)	0.174
Score vitamin E ATE (mg)	1.19(0.03)	1.13(0.03)	0.116
Score calcium (mg)	1.33(0.03)	1.19(0.03)	< 0.001
Score magnesium (mg)	1.26(0.03)	1.12(0.03)	< 0.001
Score zinc (mg)	1.19(0.03)	1.02(0.03)	< 0.001
Score copper (mg)	1.08(0.03)	0.99(0.03)	0.017
Score selenium (mcg)	1.26(0.03)	1.15(0.03)	0.005
Score total fat (g)	0.85(0.03)	0.94(0.03)	0.018
Score iron (mg)	0.87(0.03)	1.05(0.03)	< 0.001
Score total PA (MET)	1.39(0.03)	1.38(0.03)	0.804
Score alcohol (g)	0.82(0.02)	0.88(0.01)	0.003
Score BMI (kg/m^2^)	0.80(0.03)	0.71(0.03)	0.025
Score cotinine (ng/ml)	1.29(0.04)	1.15(0.04)	0.004
Dietary OBS	17.93(0.27)	16.74(0.23)	< 0.001
Lifestyle OBS	4.01(0.07)	3.74(0.06)	0.002
OBS	21.95(0.29)	20.48(0.26)	< 0.001

ATE, alpha-tocopherol equivalents; BMI, body mass index; MET, metabolic equivalent of task; PA, physical activity; OBS, oxidative balance score; RE, retinol equivalents.

Based on the calculation methodology of OBS, dietary and lifestyle scores were calculated and compared between premenopausal and postmenopausal women (Table 2). Postmenopausal women scoring lower in nutrient adequacy (e.g., dietary fiber, B vitamins, minerals) but higher in fat intake score (*p* = 0.02) and iron score (*p* < 0.001). The OBS was significantly lower in postmenopausal women (20.48 ± 0.26 vs. 21.95 ± 0.29, *p* < 0.001), driven by both dietary (16.74 ± 0.23 vs. 17.93 ± 0.27, *p* < 0.001) and lifestyle components (3.74 ± 0.06 vs. 4.01 ± 0.07, *p* = 0.002).

### 3.2 Association between OBS and the menopause

First, RCS analysis was employed to investigate whether there was a non-linear relationship between OBS and menopause. OBS knots were selected ranging from 3 to 8, and the non-linear *p-*values showed no statistical significance, indicating that there was no non-linear relationship between OBS and menopause (Supplementary Table 2). When utilizing three knots for OBS, which corresponds to the minimum AIC value, an overall *p-*value of 0.005 was obtained (Supplementary Figure 1). These results suggest that there was a correlation between OBS and the odds of menopause, but no non-linear relationship existed.

After multiple adjustments, OBS, dietary OBS, and lifestyle OBS (as continuous variables) demonstrated a negative association with the odds of menopause (*p* < 0.050, Table 3). When OBS, dietary OBS, and lifestyle OBS were grouped by quartiles, OBS, dietary OBS, and lifestyle OBS still demonstrated a negative association with the odds of menopause (*p* for trend < 0.050, Table 3).

**Table 3:** Associations between OBS and its components and menopausal status.

**Variable**	**Model**	**Continuous OR (95% CI)**	***p*-value**	**Quartile analysis OR (95% CI) by quartile**	***p*-trend**
OBS	Crude	0.97 (0.96, 0.98)	< 0.001	Q1: Ref	< 0.001
				Q2: 0.73 (0.55, 0.97)	
				Q3: 0.71 (0.53, 0.95)	
				Q4: 0.54 (0.41, 0.71)	
	Model 1^*^	0.95 (0.93, 0.97)	< 0.001	Q1: Ref	< 0.001
				Q2: 0.68 (0.46, 1.01)	
				Q3: 0.61 (0.41, 0.89)	
				Q4: 0.39 (0.25, 0.60)	
	Model 2^†^	0.97 (0.94, 0.99)	0.010	Q1: Ref	0.009
				Q2: 0.76 (0.51, 1.14)	
				Q3: 0.73 (0.47, 1.12)	
				Q4: 0.51 (0.31, 0.83)	
Dietary OBS	Crude	0.97 (0.96, 0.99)	< 0.001	Q1: Ref	< 0.001
				Q2: 0.73 (0.55, 0.97)	
				Q3: 0.71 (0.53, 0.95)	
				Q4: 0.54 (0.41, 0.71)	
	Model 1^*^	0.96 (0.93, 0.98)	< 0.001	Q1: Ref	< 0.001
				Q2: 0.68 (0.46, 1.01)	
				Q3: 0.61 (0.41, 0.89)	
				Q4: 0.39 (0.25, 0.60)	
	Model 2^†^	0.97 (0.95, 1.00)	0.040	Q1: Ref	0.009
				Q2: 0.76 (0.51, 1.14)	
				Q3: 0.73 (0.47, 1.12)	
				Q4: 0.51 (0.31, 0.83)	
Lifestyle OBS	Crude	0.90 (0.84, 0.96)	0.002	Q1: Ref	0.004
				Q2: 0.79 (0.61, 1.03)	
				Q3: 0.80 (0.60, 1.05)	
				Q4: 0.64 (0.47, 0.86)	
	Model 1^*^	0.88 (0.78, 0.98)	0.020	Q1: Ref	0.011
				Q2: 0.77 (0.53, 1.13)	
				Q3: 0.74 (0.51, 1.08)	
				Q4: 0.50 (0.31, 0.83)	
	Model 2^†^	0.88 (0.79, 0.99)	0.040	Q1: Ref	0.027
				Q2: 0.78 (0.54, 1.14)	
				Q3: 0.76 (0.51, 1.12)	
				Q4: 0.53 (0.32, 0.89)	

^*^Model 1: Adjusted for age, race, poverty-income ratio (PIR), education level, marital status, body mass index, smoking status, and alcohol consumption. ^†^Model 2: Model 1 + additional adjustment for hypertension, cardiovascular disease, diabetes mellitus, hyperlipidemia, fasting blood glucose, and daily energy intake. OR, odds ratio; 95% CI, confidence interval; OBS, Oxidative Balance Score.

In addition, multicollinearity of the covariates in Model 2 was also assessed (Supplementary Table 3). The adjusted VIF values for all covariates were < 2, significantly below the threshold of 5, indicating the absence of substantial multicollinearity (Supplementary Table 3).

### 3.3 Sensitivity analysis

To evaluate the reliability and robustness of the association between OBS and the menopause under different scenarios, sensitivity analysis and stratified analysis were conducted further. Lifestyle and dietary patterns may be subject to period effects, consequently modifying the OBS over time. Additionally, the survey years included in this study span from 2003 to 2020, encompassing a total of nine survey year cycles over a period of 17 years. Therefore, in order to assess the robustness of the association between OBS and menopause, different survey year cycles were systematically excluded in the sensitivity analysis. As a result, even after excluding any single survey year cycle, the association between OBS and menopausal odds remained significant (*p* < 0.050, Table 4). To assess whether the model exhibited overfitting, the AUCs of model 2 were calculated in the sensitivity analysis. All the AUC values exceeded 0.860 with high stability (SD = 0.0046 and all the ΔAUC values < 0.15) indicated that the model had a good generalization ability. Moreover, the residual plots of model 2, applied in sensitivity analysis across different datasets, showed that the residuals were randomly distributed above and below zero, indicating a good model fit (Supplementary Figure 2).

**Table 4:** Association between OBS and menopause with the exclusion of each survey year cycle.

**Excluded year cycle**	**Excluded participant number (percentage)**	**Q1**	**Q2**		**Q3**		**Q4**		***p* for trend**	**AUC**
			**OR (95%CI)**	***p*** **value**	**OR (95%CI)**	***p*** **value**	**OR (95%CI)**	***p*** **value**		
2003–2004	268 (10%)	Ref.	0.67 (0.45,1.01)	0.054	0.71 (0.44,1.15)	0.166	0.44 (0.26,0.74)	0.002	0.006	0.873
2005–2006	267 (9%)	Ref.	0.79 (0.50,1.23)	0.289	0.79 (0.50,1.26)	0.320	0.55 (0.32,0.94)	0.028	0.037	0.880
2007–2008	316 (11%)	Ref.	0.69 (0.44,1.08)	0.102	0.59 (0.36,0.95)	0.032	0.43 (0.24,0.76)	0.004	0.006	0.875
2009–2010	358 (13%)	Ref.	0.83 (0.54,1.26)	0.372	0.86 (0.55,1.35)	0.507	0.56 (0.32,0.93)	0.027	0.037	0.872
2011–2012	288 (10%)	Ref.	0.80 (0.52,1.22)	0.297	0.78 (0.50,1.20)	0.256	0.51 (0.30,0.86)	0.013	0.016	0.877
2013–2014	359 (13%)	Ref.	0.75 (0.49,1.16)	0.195	0.68 (0.43,1.08)	0.103	0.46 (0.28,0.77)	0.004	0.004	0.865
2015–2016	311 (11%)	Ref.	0.87 (0.57,1.32)	0.502	0.71 (0.46,1.10)	0.128	0.60 (0.37,0.98)	0.043	0.032	0.869
2017–2018	237 (8%)	Ref.	0.72 (0.47,1.09)	0.115	0.73 (0.47,1.14)	0.167	0.52 (0.31,0.88)	0.014	0.022	0.869
2019–2020	409 (15%)	Ref.	0.76 (0.50,1.15)	0.196	0.75 (0.48,1.17)	0.200	0.51 (0.31,0.84)	0.009	0.012	0.873

The logistic regression models were adjusted by age, race, PIR, education background, single status, body mass index, smoking behavior, alcohol consumption, hypertension, cardiovascular disease, diabetes mellitus, hyperlipidaemia, fast blood glucose, and daily energy intake. OR, odds ratio; 95% CI, confidence interval.

Moreover, the stratified analysis was further conducted to identify effect differences across various populations or subgroups. However, the significant association between OBS and menopause status was only observed in the 40–44 year cohort (Q3: OR = 0.351, 95% CI 0.149–0.825; *p* for trend = 0.02), while other stratified analyses did not yield significant results for any of the other subgroups (Supplementary Table 4). This suggests that the age range of 40–44 years may represent a critical time window for the association between OBS and menopause. Preliminary analyses suggest that the association between OBS and the odds of menopause was influenced by both obesity (p for interaction = 0.029) and hypertension (p for interaction = 0.032, Supplementary Table 4). To control the false discovery rate (FDR) when conducting multiple hypothesis tests, the Benjamini-Hochberg adjustment was applied to the *p-*values for interaction. After FDR correction, all interaction *p-*values exceeded the significance threshold (FDR-adjusted *p* > 0.10), with the adjusted *p-*values for obesity (adjusted p for interaction = 0.983) and hypertension (adjusted p for interaction = 0.547) suggesting the presence of uncorrected false positive results (Supplementary Table 4).

### 3.4 Comparison of OBS and its components across premenopausal and various postmenopausal phases

**Figure 1:**
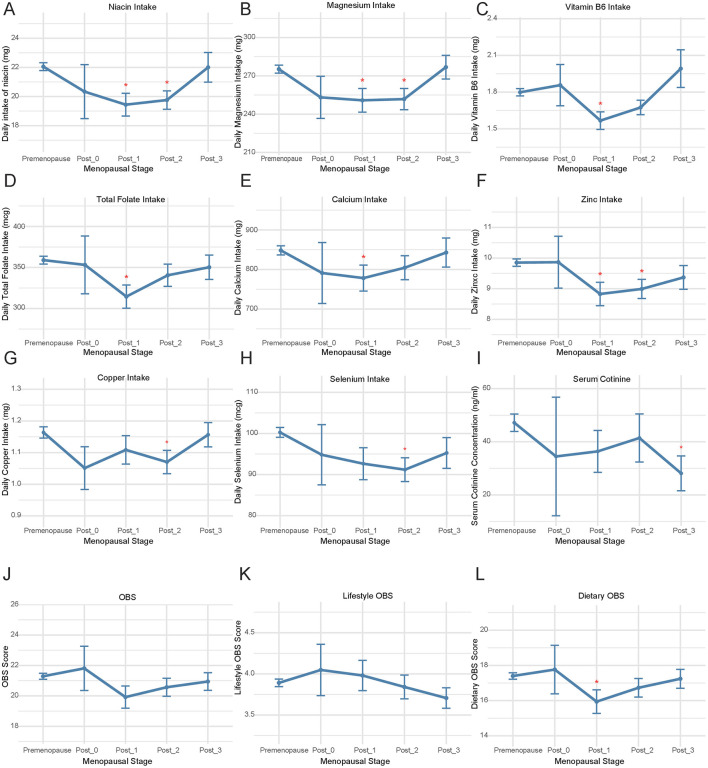
The comparison of OBS and its components during menopausal transition. Comparative analysis was performed between premenopausal women and four postmenopausal subgroups stratified by years since menopause onset: <1 year (Post_0), 1–2 years (Post_1), 2–3 years (Post_2), and 3-4 years (Post_3) for **(A)** niacin intake, **(B)** magnesium intake, **(C)** vitamin B6 intake; **(D)** total folate intake; **(E)** calcium intake; **(F)** zinc intake, **(G)** copper intake, **(H)** selenium intake, **(I)** serum cotinine levels, **(J)** OBS, **(K)** Lifestyle OBS, **(L)** Dietary OBS. OBS, Oxidative Balance Score.

The comparison of OBS and its components was conducted between premenopausal individuals and those in the postmenopausal phases of < 1 year (Post_0), 1–2 years (Post_1), 2–3 years (Post_2), and 3–4 years (Post_3) (Figure 1, Supplementary Table 5). In the population of Post_1, compared to premenopausal individuals, there was a significant decrease in the intake of niacin (*p* = 0.002, Figure 1A), magnesium (*p* = 0.014, Figure 1B), vitamin B6 (*p* = 0.003, Figure 1C), total folate (*p* = 0.004, Figure 1C), calcium (*p* = 0.047, Figure 1E), and zinc (*p* = 0.012, Figure 1F). In the Post_2 population, there were significant decreases in the intake of niacin (*p* = 0.001, Figure 1A), magnesium (*p* = 0.009, Figure 1B), zinc (*p* = 0.011, Figure 1F), iron (*p* = 0.027, Supplementary Table 5), copper (*p* = 0.024, Figure 1G), and selenium (*p* = 0.004, Figure 1H) compared to premenopausal individuals. Compared to premenopausal levels, the components that showed decreased intake in the Post_1 and Post_2 populations did not exhibit significant differences in the Post_3 population. Furthermore, compared to premenopausal levels, serum cotinine showed a significant decline in the Post_3 phase (*p* = 0.011, Figure 1I), and there was a decrease in dietary OBS during the Post_1 phase (*p* = 0.038, Figure 1L). Although cross-sectional studies cannot establish causality, these findings suggest that menopausal status may not be a significant influencing factor for OBS, as no substantial changes in OBS were observed over several years before and after menopause (Figures 1J, K). Women's lifestyle and dietary habits do not undergo significant changes in the short term due to menopause.

Furthermore, linear regression was employed to investigate the relationship between the duration of time since menopause and OBS. It was found that OBS decreases with increasing years post-menopause after adjusting for race, PIR, education, marital status, BMI, smoking, alcohol use, hypertension, CVD, DM, hyperlipidemia, daily energy intake, and fasting glucose levels (*p* < 0.001, Supplementary Figure 3A). The linear relationship between age and OBS was also analyzed using the same methodology. Similar to the correlation trend observed between OBS and the years post-menopause, in women aged 40–60, OBS gradually declines with increasing age (*p* < 0.001, Supplementary Figure 3B).

### 3.5 Establishment of classification models

**Figure 2:**
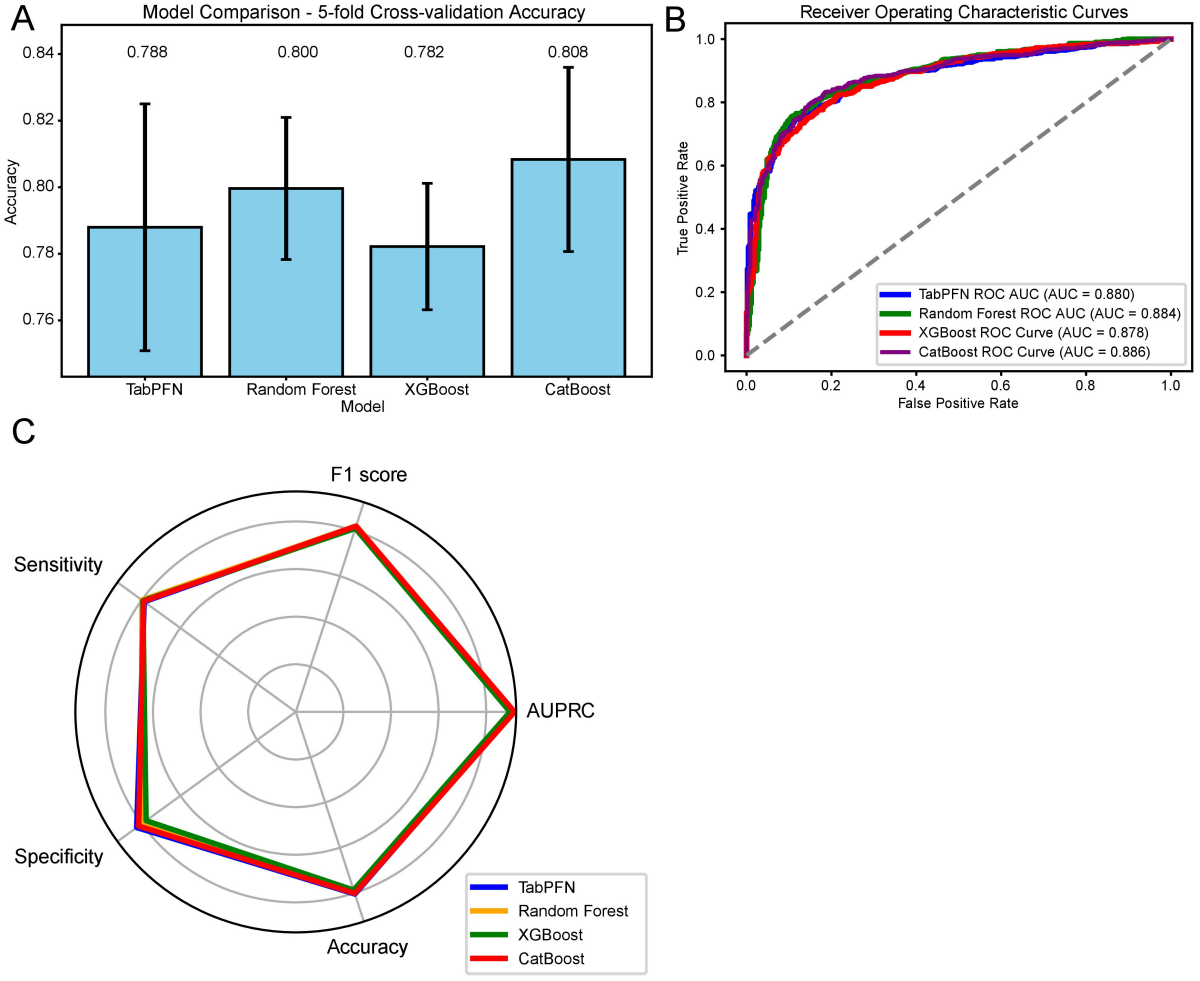
Machine learning model performance in menopausal status classification based on OBS and Its Components. **(A)** Five-fold cross-validation accuracy of the classification models in the training set. **(B)** ROC curves with AUC values for the classification models in the testing set. **(C)** Comprehensive evaluation metrics, including F1 score, sensitivity, specificity, accuracy, and AUPRC for the classification models in the testing set. OBS, Oxidative Balance Score; ROC, Receiver Operating Characteristic; AUC, Area Under the Curve; AUPRC, Area Under the Precision-Recall Curve; RF, Random Forest; TabPFN, Tabular Prior-Data Fitted Networks; CV, Cross-Validation.

The classification models for menopausal status based on OBS and its components were established using machine learning algorithms. First, the data were split into a training dataset and a test dataset in a 1:1 ratio, with the random state set to 42. Confounding factors from the multivariate logistic regression were included, and classification models were established using TabPFN, Random Forest, XGBoost, and CatBoost. In the training set, the mean accuracy obtained through 5-fold cross-validation was as follows: CatBoost 0.808, RF 0.800, TabPFN 0.788, and XGBoost 0.782 (Figure 2A, Supplementary Table 6). In the testing set, the menopause classification models developed using TabFPN, Random Forest, CatBoost, and XGBoost achieved an AUC of 0.880, 0.884, 0.886, and 0.878, respectively (Figure 2B). Beside AUC, F1 score, AUPRC, sensitivity, specificity, and accuracy of four classification models also reached a good level (Figure 2C, Supplementary Table 7). The accuracy of the testing set ranges from 0.787 to 0.805, and a delta of < 0.15 in accuracy during 5-fold cross-validation among the four models in the training set suggested that there was no evidence of overfitting of the classification models.

**Figure 3:**
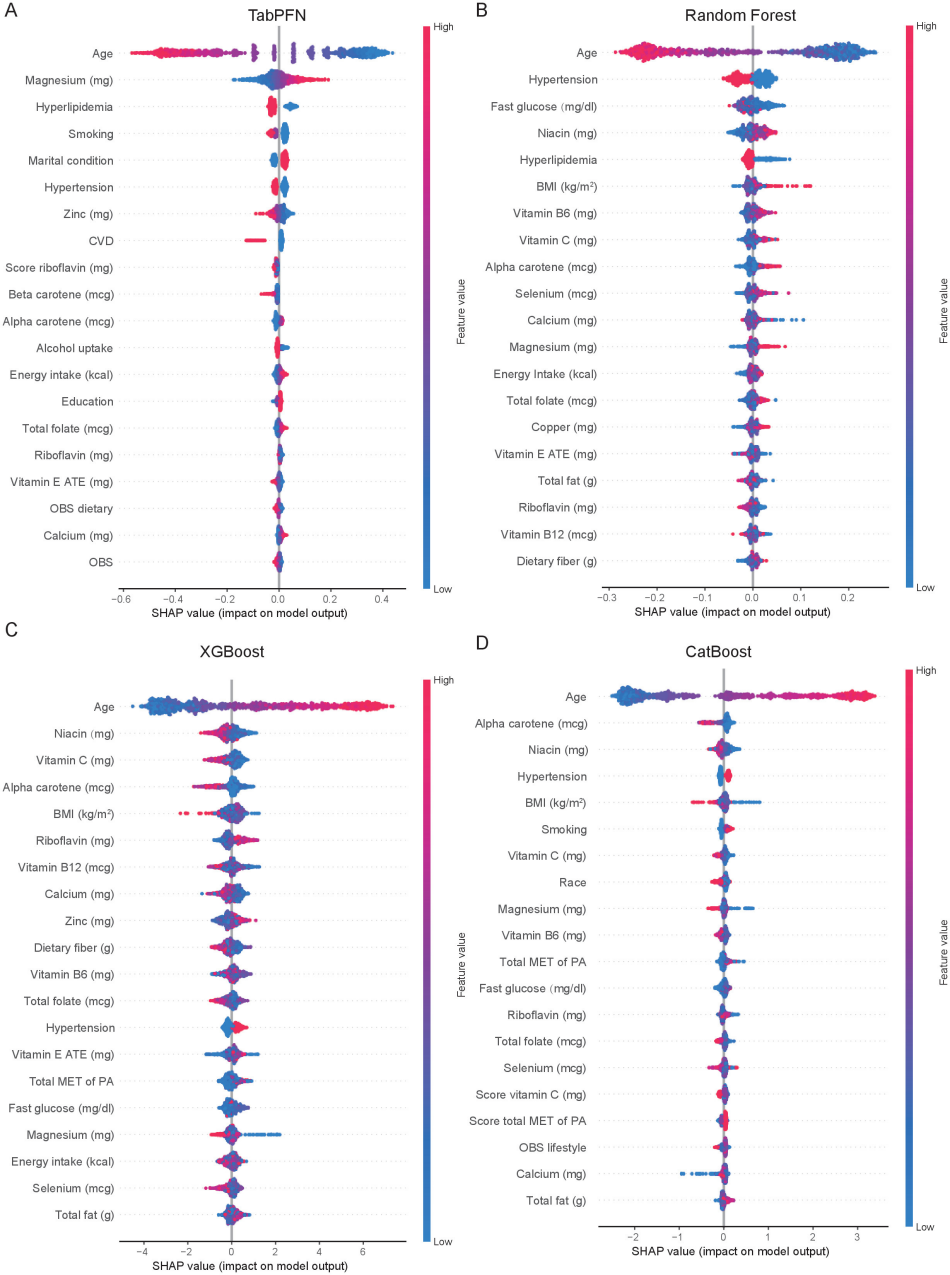
Comparative feature importance analysis of machine learning models for menopausal status classification. Feature importance rankings across TabPFN model **(A)**, Random Forest **(B)**, XGBoost **(C)**, and CatBoost **(D)** for classifying menopausal status, as quantified by SHAP values. Variables are ordered by their mean absolute SHAP impact on model output. OBS, Oxidative Balance Score; SHAP, SHapley Additive exPlanations; CVD, Cardiovascular Disease; BMI, Body Mass Index; ATE, Alpha-Tocopherol Equivalents (for Vitamin E measurement); MET, Metabolic Equivalent of Task; PA, Physical Activity; TabPFN, Tabular Prior-Data Fitted Networks; XGBoost, eXtreme Gradient Boosting.

The SHAP values, which quantify the impact of individual features on the model's output, were displayed in the Figure 3. Ranked by the importance of features, age, magnesium, hyperlipidemia, smoking, and hypertension were the top 5 features in the TabPFN model (Figure 3A). Age, hypertension, fasting glucose, niacin, and hperlipidemia were most influential features in the Random Forest model (Figure 3B). Age, niacin, vitamin C, alpha carotene, and BMI were key features in the XGBoost model (Figure 3C). Age, alpha carotene, niacin, hypertension, and BMI were important features in the CatBoost model (Figure 3D). Age is consistently the most influential feature across all models, followed by other health-related variables such as hypertension, BMI, and specific nutrient intakes (e.g., magnesium, niacin, vitamin C).

## 4 Discussion

After adjusting for various confounding factors, the OBS showed an inverse relationship with menopause (OR: 0.97, 95% CI: 0.94–0.99, *P* = 0.01). When the OBS was divided into quartiles, the significant association with menopause persisted (*P* for trend = 0.009). This relationship remained significant even after excluding data from any individual survey year cycles (*P* for trend < 0.05). Moreover, the intake of magnesium, zinc, niacin, and vitamin B6 showed a decline in the early postmenopausal period. Finally, classification models were established using four machine learning algorithms. The performance of all models was relatively good with an AUC of ≥ 0.878.

The stratified analyses indicated a significant association between OBS and menopause only in the 40–44 age group, which might preliminarily suggest a timeframe for lifestyle and dietary adjustments in perimenopausal women. However, no robust subgroup-specific associations were observed after multiplicity correction. A possible reason for this is the reduced sample size in each subgroup after stratification. Future research should aim to expand the sample size and take measures to minimize false positives.

Age consistently ranks as the most important feature in all four classification models for menopausal status. The menopause is a natural biological process associated with the depletion of ovarian follicular function, generally occurring in women aged 45–55 years (20). Age represents the foremost intrinsic factor influencing ovarian functionality and fertility. With advancing age, women experience a progressive decline in the quantity of oocytes, and these oocytes remain constantly exposed to ROS. In ovarian tissue, normal levels of ROS play a crucial role in regulating follicular growth, angiogenesis, and steroid hormone synthesis. OS results in a decline in the quantity and quality of oocytes, mediating changes in genetic material, signaling pathways, transcription factors, and the ovarian microenvironment, which in turn leads to abnormal apoptosis of granulosa cells, dysregulated meiosis, and a reduction in mitochondrial DNA and other alterations, thereby accelerating the ovarian aging process (21). DNA damage and changes in genetic material mediated by OS include apoptosis, dysfunction of mitochondrial DNA, abnormalities in meiosis, and telomere shortening (22). Elevated levels of ROS are often associated with chromosomal instability or abnormalities, spindle defects, decreased mitochondrial function, and telomere shortening in oocytes of advanced maternal age (over 35 years) (23). The reduced telomerase activity and telomere dysfunction in oocytes are associated with diminished reproductive capacity and infertility in women of advanced maternal age. The impact of ROS on ovarian function may represent a potential mechanism of the association between menopause and OS. Besides, with aging, there is an increase in the generation of ROS and a reduction in the effectiveness of antioxidant systems, which is consistent with the linear regression findings in this study that reveal a negative relationship between OBS and age. The progressive ROS accumulation of ROS with age, combined with the accelerated production of ROS and the weakened capacity of the antioxidant defense system in the elderly, results in enhanced OS damage at the cellular level (24). Mitochondrial dysfunction and biomolecular damage resulting from aging also accumulate in ovarian cells as age increases, contributing to the decline in ovarian function (24).

Daily magnesium intake is an important feature in all four classification models for menopausal status. Moreover, based on this study, magnesium intake decreased in the postmenopausal 1–2 years and (250.83 ± 9.26 mg, *p* = 0.014) and in the postmenopausal 2–3 years (251.78 ± 8.30 mg, *p* = 0.009) compared to the premenopausal level of 275.24 ± 3.13 mg. Magnesium exhibits a mild antioxidant effect *in vivo* (25). Aging is an additional risk factor for inadequate magnesium intake, with a gradual decline in magnesium consumption observed as individuals age (26). A deficiency in magnesium is associated with a rise in the levels of ROS, an increase in hydrogen peroxide production, and an upsurge in the production of superoxide anions by inflammatory cells (27). Moreover, magnesium insufficiency not only exacerbates OS but also reduces the effectiveness of antioxidant defense systems (28). The findings of this study on epidemiology and machine learning suggest that attention should be paid to perimenopausal women regarding magnesium intake.

Postmenopausal women exhibited significantly reduced zinc intake compared to premenopausal levels (premenopausal: 9.85 ± 0.12 mg/day; postmenopausal 1–2 years: 8.83 ± 0.38 mg/day, *p* = 0.012; postmenopausal 2–3 years: 8.99 ± 0.31 mg/day, *p* = 0.011). This decline is clinically noteworthy given zinc's prominence as a top-ranked feature in both TabPFN and XGBoost classification models. The apparent adequacy of zinc intake (≥8 mg/d) in perimenopausal populations may not translate to sufficient systemic availability as only 10% to 12% of zinc is effectively absorbed due to a diet with low bioavailability (29). Zinc deficiency impairs the catalytic function of numerous antioxidant enzymes, notably copper/zinc superoxide dismutase (SOD1), compromising its critical role in preventing oxidative DNA damages (30).

Niacin, an antioxidant, is ranked as the second, third, and fourth most important feature in the XGBoost, RF, and CatBoost models, respectively. The menopausal transition was associated with a 10–12% reduction in niacin intake, a finding reinforced by its consistent importance as a classification variable across machine learning methods. Niacin, commonly referred to as B3, serves as a precursor for the synthesis of the pyridine coenzymes nicotinamide adenine dinucleotide (NAD) and nicotinamide adenine dinucleotide phosphate (NADP). A deficiency of niacin may limit the NAD+/NADH pool, thereby compromising sirtuin-mediated stress response pathways and creating a permissive environment for the accumulation of oxidative damage (31). In animal models of premature ovarian insufficiency, administration of niacin was found to suppress follicular apoptosis in adverse conditions and markedly decrease apoptosis in cumulus cells (32). Additionally, there was an observed increase in the number of developing follicles following niacin treatment (32). Niacin enhances the number of healthy antral follicles and corpora lutea in rats with polycystic ovary syndrome, while simultaneously reducing the quantity of cystic follicles and the thickness of the thecal layer surrounding the follicles (33).

A significant reduction in vitamin B6 levels was observed in early postmenopausal women (*p* = 0.003), with consistent identification as a high-importance feature across multiple machine learning algorithms (XGBoost, RF, and CatBoost). In addition to its role as a cofactor for various enzyme-catalyzed biochemical reactions, vitamin B6 also functions as a scavenger of ROS, a metal chelator, and a chaperone in the enzyme folding process (34). This nutritional alteration carries particular clinical relevance given vitamin B6's essential role in homocysteine metabolism. The observed decline during menopausal transition may compromise the transsulfuration pathway, potentially elevating circulating homocysteine levels and thereby contributing to the increased cardiovascular risk profile characteristic of the menopausal transition (35).

The production of ROS can be induced by various factors, including heavy metals, tobacco smoke, drugs, exogenous substances, pollutants, and radiation. Oxidative stress resulting from poor lifestyle habits is associated with various age-related diseases (36). Smoking plays a significant role in the performance of the models established using TabPFN and CatBoost. Both current and former smokers are at an increased risk of experiencing earlier menopause (37). The presence of stable pro-oxidants in tobacco smoke can lead to a direct increase in ROS within the body. Furthermore, substances like nicotine and tar found in tobacco can exhaust protective antioxidants, thereby contributing to the development of oxidative stress (24). Any level of smoke exposure can induce oxidative stress damage to the ovaries (24).

Hypertension emerged as a crucial feature influencing the menopause prediction models established using both CatBoost and TabPFN. OS may be one of the mechanisms involved in the development of hypertension (38). Moreover, antioxidants such as alpha carotene and vitamin C contribute to the performance of the model predictions. This further confirms the correlation between OBS and the odds of menopause occurrence.

The limitations of this study highlight that our cross-sectional data can indicate a correlation between OBS (observational biomarker) and menopause, but it cannot infer a causal relationship. To determine whether menopause also affects OBS, longitudinal studies are needed to track the changes in OBS and menopause over time, as well as their temporal relationships. Alternatively, utilizing time series data could help validate the causal relationships between the different variables. One notable limitation is the inability to validate the model externally using independent datasets from other populations, which restricts the generalizability of our findings. The lack of comparable external data prevents further confirmation of the model's robustness and reproducibility across different settings. Future studies should prioritize multicenter collaborations to obtain diverse validation cohorts and strengthen the clinical applicability of the model.

This study identified the correlation between OBS and menopause using nationwide cross-sectional sampling data. The robustness of this correlation was confirmed through sensitivity analysis. Stratified analysis might indicate that the sensitive time window for the correlation between OBS and menopausal status is between the ages of 40 and 44. Additionally, a decrease in the intake of magnesium, zinc, niacin, and vitamin B6 was observed during menopausal transition. Finally, four different machine learning algorithms were employed to establish well-performing menopause classification models based on epidemiological information and OBS along with its components, confirming the roles of magnesium, zinc, niacin, and vitamin B6 in the perimenopausal period. However, the cross-sectional nature of the study restricts the capacity to establish causal inferences. This study, while unable to confirm a causal relationship between OS and menopause, presents preliminary findings on the enhancement of ovarian function through dietary and behavioral modifications.

## 5 Conclusion

The results of this study imply a reverse association between OBS and menopause and the time window and content for dietary and lifestyle improvements during the menopausal transition.

## Data Availability

The original contributions presented in the study are included in the article/Supplementary material, further inquiries can be directed to the corresponding author.
