# Supplementary material for: Oxidative balance score and menopausal status: insights from epidemiological analysis and machine learning models
Source: Front Nutr. 2025 May 20;12:1586606. doi: 10.3389/fnut.2025.1586606 (PMC12129807; doi:10.3389/fnut.2025.1586606)
Supplement: Supplementary file 1 [file Data_Sheet_1.docx]

Supplementary Material

# Supplementary Tables

Supplementary Table 1. The approved protocol number of each survey year cycle.

| Survey year cycle | Approved protocol number |
| --- | --- |
| NHANES 2003-2004 | Protocol #98-12 |
| NHANES 2005-2006 | Protocol #2005-06 |
| NHANES 2007-2008 and NHANES 2009-2010 | Continuation of Protocol #2005-06 |
| NHANES 2011-2012 | Protocol #2011-17 |
| NHANES 2013-2014, NHANES 2015-2016, and NHANES 2017-2018 (Effective through 26 October 2017) | Continuation of Protocol #2011-17 |
| NHANES 2017-2018 (Effective beginning 26 October 2017) and NHANES 2019-2020 | Protocol #2018-01 |

NHANES: National Health and Nutrition Examination Survey; OBS: Oxidative Balance Score.

Supplementary Table 2. Summary of AIC values and p-values for non-linearity across different OBS knot selections.

| **OBS knot** | **AIC** | ***p* nonlinear** | **Significance** |
| --- | --- | --- | --- |
| 3 | 2545.075 | 0.6959404 | >= 0.05 |
| 4 | 2545.653 | 0.4555654 | >= 0.05 |
| 5 | 2546.713 | 0.4736707 | >= 0.05 |
| 6 | 2548.891 | 0.6747315 | >= 0.05 |
| 7 | 2549.086 | 0.5302457 | >= 0.05 |
| 8 | 2547.968 | 0.3006573 | >= 0.05 |

OBS: Oxidative Balance Score; AIC: Akaike Information Criterion; p: p-value.

Supplementary Table 3. Summary of GVIF, DF, and adjusted GVIF values for covariates.

| Variable | GVIF | Df | GVIF^(1/(2*Df)) |
| --- | --- | --- | --- |
| OBS | 1.940077 | 1 | 1.392867 |
| Age | 1.229605 | 1 | 1.108876 |
| Race | 2.796662 | 3 | 1.186971 |
| PIR | 1.821667 | 1 | 1.349691 |
| Eduation | 2.499129 | 2 | 1.257324 |
| Single status | 1.390800 | 1 | 1.179322 |
| BMI | 1.708594 | 1 | 1.307132 |
| Smoking | 2.487170 | 2 | 1.255817 |
| Alcohol uptake | 1.788641 | 2 | 1.156461 |
| Hypertension | 1.728979 | 1 | 1.314906 |
| CVD | 1.140773 | 1 | 1.068070 |
| DM | 3.128276 | 1 | 1.768693 |
| Hyperlipidemia | 1.296888 | 1 | 1.138810 |
| Daily energy intake | 1.644832 | 1 | 1.282510 |
| Fast glucose | 3.177346 | 1 | 1.782511 |

GVIF: Generalized Variance Inflation Factor; Df: Degrees of Freedom; OBS: Observational Behavioral Scale; PIR: Poverty Income Ratio; CVD: Cardiovascular Disease; DM: Diabetes Mellitus.

Supplementary Table 4. Stratified analysis of the association between OBS and menopausal odds

| Variables | Q1 | Q2 | Q3 | Q4 | *p* for trend | *p* for interaction | Benjamini-Hochberg Adjusted *p* for interaction |
| --- | --- | --- | --- | --- | --- | --- | --- |
| Age group |  |  |  |  |  | 0.663 | 0.365 |
| 40-44 | Ref. | 0.692(0.324,1.481) | 0.351(0.149,0.825) | 0.395(0.142,1.098) | 0.02 |  |  |
| 45-49 | Ref. | 0.834(0.459,1.515) | 0.883(0.453,1.721) | 0.643(0.296,1.397) | 0.327 |  |  |
| 50-54 | Ref. | 0.777(0.389,1.549) | 1.070(0.505,2.265) | 0.505(0.202,1.265) | 0.218 |  |  |
| 55-60 | Ref. | 1.073(0.178,6.454) | 1.454(0.250,8.459) | 1.233(0.253,6.009) | 0.77 |  |  |
| Race |  |  |  |  |  | 0.103 | 0.999 |
| Non-Hispanic white | Ref. | 0.848(0.544,1.322) | 1.153(0.699,1.902) | 0.978(0.581,1.646) | 0.778 |  |  |
| Non-Hispanic black | Ref. | 1.223(0.700,2.137) | 1.091(0.645,1.846) | 2.169(1.069,4.404) | 0.113 |  |  |
| Mexican American | Ref. | 1.755(0.936, 3.291) | 1.791(0.728, 4.406) | 1.055(0.433, 2.572) | 0.996 |  |  |
| Other | Ref. | 0.967(0.455,2.056) | 0.637(0.303,1.341) | 0.879(0.356,2.170) | 0.569 |  |  |
| PIR |  |  |  |  |  | 0.999 | 0.365 |
| 1.3-3.5 | Ref. | 1.035(0.649,1.650) | 1.308(0.734,2.329) | 1.355(0.735,2.497) | 0.263 |  |  |
| <=1.3 | Ref. | 0.823(0.487,1.392) | 0.794(0.428,1.473) | 0.688(0.347,1.367) | 0.282 |  |  |
| >3.5 | Ref. | 0.945(0.551, 1.619) | 1.054(0.590, 1.881) | 0.963(0.528, 1.757) | 0.993 |  |  |
| Education |  |  |  |  |  | 0.152 | 0.547 |
| <9 years | Ref. | 1.514(0.480, 4.776) | 2.205(0.403, 12.080) | 2.489(0.459, 13.491) | 0.245 |  |  |
| 9-12 years | Ref. | 1.385(0.883,2.174) | 1.001(0.551,1.818) | 1.155(0.554,2.406) | 0.916 |  |  |
| >12 years | Ref. | 0.705(0.455,1.092) | 0.992(0.623,1.578) | 0.884(0.535,1.460) | 0.87 |  |  |
| Single status |  |  |  |  |  | 0.319 | 0.192 |
| Single | Ref. | 0.828(0.542,1.264) | 0.845(0.504,1.417) | 0.939(0.466,1.891) | 0.901 |  |  |
| Not single | Ref. | 0.967(0.640,1.459) | 1.191(0.743,1.907) | 1.012(0.619,1.657) | 0.796 |  |  |
| Obesity |  |  |  |  |  | 0.029 | 0.983 |
| Underweight/normal | Ref. | 1.148(0.587, 2.243) | 2.516(1.213, 5.220) | 1.256(0.585, 2.698) | 0.479 |  |  |
| Obesity | Ref. | 0.913(0.602,1.386) | 0.859(0.493,1.499) | 0.834(0.495,1.406) | 0.497 |  |  |
| Overweight | Ref. | 0.907(0.467,1.761) | 0.773(0.402,1.486) | 1.382(0.655,2.917) | 0.443 |  |  |
| Smoking |  |  |  |  |  | 0.901 | 0.983 |
| Never | Ref. | 1.063(0.690,1.637) | 1.142(0.688,1.896) | 1.050(0.613,1.797) | 0.869 |  |  |
| Former | Ref. | 1.001(0.491,2.042) | 1.432(0.618,3.321) | 1.282(0.563,2.922) | 0.422 |  |  |
| Now | Ref. | 0.684(0.378,1.237) | 0.741(0.360,1.524) | 0.933(0.351,2.480) | 0.608 |  |  |
| Alcohol uptake |  |  |  |  |  | 0.881 | 0.192 |
| Never | Ref. | 1.065(0.491,2.310) | 0.879(0.330,2.340) | 1.070(0.381,3.005) | 0.991 |  |  |
| Former | Ref. | 0.551(0.258,1.177) | 0.539(0.227,1.276) | 0.427(0.144,1.267) | 0.133 |  |  |
| Now | Ref. | 0.949(0.647,1.391) | 1.230(0.805,1.881) | 1.111(0.698,1.769) | 0.45 |  |  |
| Hypertension |  |  |  |  |  | 0.032 | 0.547 |
| Yes | Ref. | 0.791(0.461,1.356) | 0.641(0.358,1.147) | 1.042(0.517,2.100) | 0.981 |  |  |
| No | Ref. | 1.035(0.683,1.569) | 1.435(0.907,2.269) | 1.048(0.630,1.743) | 0.647 |  |  |
| CVD |  |  |  |  |  | 0.305 | 0.365 |
| No | Ref. | 0.957(0.688,1.332) | 1.132(0.788,1.626) | 1.046(0.704,1.555) | 0.64 |  |  |
| Yes | Ref. | 0.789(0.184, 3.374) | 0.370(0.083, 1.655) | 0.698(0.088, 5.518) | 0.326 |  |  |
| DM |  |  |  |  |  | 0.122 | 0.939 |
| No | Ref. | 0.929(0.660,1.308) | 1.163(0.789,1.716) | 1.031(0.687,1.546) | 0.642 |  |  |
| Yes | Ref. | 1.240(0.612,2.512) | 0.864(0.351,2.129) | 1.224(0.416,3.603) | 0.873 |  |  |
| Hyperlipidemia |  |  |  |  |  | 0.704 | 0.939 |
| Yes | Ref. | 0.912(0.644,1.291) | 1.148(0.782,1.685) | 1.114(0.720,1.724) | 0.419 |  |  |
| No | Ref. | 1.108(0.548,2.238) | 0.951(0.484,1.867) | 0.828(0.361,1.899) | 0.49 |  |  |

The logistic regression models were adjusted by age, race, PIR, education background, single status, body mass index, smoking behaviour, alcohol consumption, hypertension, cardiovascular disease, diabetes mellitus, hyperlipidaemia, fast blood glucose, and daily energy intake. OR, odds ratio; 95% CI, confidence interval; PIR, poverty index ratio; CVD, cardiovascular disease; DM, diabetes mellitus.

Supplementary Table 5. Comparison of OBS and its components across premenopausal and various postmenopausal phases

| Parameter | Premenopaue (Mean±SE) | Post-0 (Mean±SE) | p (vs Premenopaue) | Post-1 (Mean±SE) | p (vs Premenopaue) | Post-2 (Mean±SE) | p (vs Premenopaue) | Post-3 (Mean±SE) | p (vs Premenopaue) |
| --- | --- | --- | --- | --- | --- | --- | --- | --- | --- |
| Anthropometrics |  |  |  |  |  |  |  |  |  |
| BMI (kg/m²) | 30.57±0.24 | 33.42±2.06 | 0.185 | 29.42±0.74 | 0.141 | 29.97±0.62 | 0.364 | 30.70±0.63 | 0.853 |
| Macronutrients |  |  |  |  |  |  |  |  |  |
| Dietary fiber (g) | 15.81±0.23 | 14.61±1.21 | 0.336 | 14.85±0.77 | 0.231 | 15.05±0.65 | 0.268 | 15.44±0.62 | 0.574 |
| Total fat (g) | 72.64±0.90 | 67.11±4.40 | 0.231 | 70.58±2.79 | 0.484 | 68.06±2.64 | 0.102 | 70.73±2.45 | 0.465 |
| Vitamins |  |  |  |  |  |  |  |  |  |
| Alpha-carotene (mcg) | 386.64±19.21 | 774.43±218.82 | 0.093 | 428.89±84.80 | 0.628 | 325.52±50.35 | 0.258 | 451.62±86.18 | 0.463 |
| Beta-carotene (mcg) | 2203.33±83.20 | 2933.50±624.64 | 0.260 | 2505.02±342.01 | 0.393 | 2333.58±218.04 | 0.577 | 2092.44±202.29 | 0.613 |
| Riboflavin (mg) | 1.87±0.02 | 2.04±0.20 | 0.393 | 1.79±0.07 | 0.307 | 1.77±0.06 | 0.140 | 1.84±0.07 | 0.726 |
| Niacin (mg) | 22.05±0.27 | 20.33±1.85 | 0.370 | 19.44±0.78* | 0.002 | 19.76±0.63* | 0.001 | 22.00±1.02 | 0.964 |
| Vitamin B6 (mg) | 1.80±0.03 | 1.86±0.17 | 0.737 | 1.57±0.07* | 0.003 | 1.67±0.06 | 0.063 | 1.99±0.15 | 0.219 |
| Total folate (mcg) | 358.97±4.78 | 353.19±35.19 | 0.872 | 314.56±14.15* | 0.004 | 340.56±13.56 | 0.202 | 350.30±14.81 | 0.578 |
| Vitamin B12 (mcg) | 4.16±0.10 | 4.73±0.73 | 0.443 | 3.67±0.25 | 0.070 | 3.86±0.21 | 0.212 | 4.50±0.34 | 0.333 |
| Vitamin C (mg) | 79.59±2.09 | 71.76±11.59 | 0.513 | 78.57±6.86 | 0.887 | 83.47±5.94 | 0.539 | 87.72±5.93 | 0.198 |
| Vitamin E (mg) | 7.76±0.14 | 7.22±0.59 | 0.388 | 7.05±0.38 | 0.083 | 7.56±0.39 | 0.631 | 7.75±0.38 | 0.986 |
| Minerals |  |  |  |  |  |  |  |  |  |
| Calcium (mg) | 848.27±11.55 | 791.12±77.06 | 0.471 | 778.27±32.90* | 0.047 | 804.53±30.32 | 0.179 | 842.85±36.74 | 0.888 |
| Magnesium (mg) | 275.24±3.13 | 253.14±16.51 | 0.202 | 250.83±9.26* | 0.014 | 251.78±8.30* | 0.009 | 276.78±9.26 | 0.875 |
| Iron (mg) | 13.15±0.16 | 13.43±1.25 | 0.824 | 12.28±0.51 | 0.108 | 12.05±0.47* | 0.027 | 12.93±0.56 | 0.707 |
| Zinc (mg) | 9.85±0.12 | 9.86±0.85 | 0.988 | 8.83±0.38* | 0.012 | 8.99±0.31* | 0.011 | 9.37±0.39 | 0.234 |
| Copper (mg) | 1.16±0.02 | 1.05±0.07 | 0.121 | 1.11±0.04 | 0.257 | 1.07±0.04* | 0.024 | 1.16±0.04 | 0.866 |
| Selenium (mcg) | 100.26±1.20 | 94.81±7.32 | 0.471 | 92.64±3.90 | 0.064 | 91.18±2.90* | 0.004 | 95.25±3.74 | 0.204 |
| Lifestyle |  |  |  |  |  |  |  |  |  |
| Alcohol (g) | 5.57±0.40 | 3.31±1.83 | 0.242 | 4.78±1.11 | 0.509 | 3.91±0.95 | 0.110 | 6.76±1.37 | 0.404 |
| PA (MET-min/week) | 2989.93±145.00 | 3864.61±2623.53 | 0.762 | 3796.56±502.44 | 0.171 | 2627.21±352.25 | 0.415 | 2956.43±407.10 | 0.947 |
| Cotinine (ng/ml) | 47.17±3.28 | 34.48±22.32 | 0.580 | 36.39±7.88 | 0.209 | 41.43±9.04 | 0.554 | 28.12±6.55 | 0.011 |
| Composite Scores |  |  |  |  |  |  |  |  |  |
| OBS | 21.29±0.20 | 21.81±1.46 | 0.726 | 19.92±0.73 | 0.073 | 20.57±0.59 | 0.249 | 20.94±0.58 | 0.576 |
| Dietary OBS | 17.40±0.18 | 17.76±1.38 | 0.796 | 15.94±0.67* | 0.038 | 16.73±0.53 | 0.231 | 17.24±0.54 | 0.780 |
| Lifestyle OBS | 3.89±0.05 | 4.05±0.31 | 0.625 | 3.98±0.18 | 0.640 | 3.84±0.14 | 0.741 | 3.71±0.12 | 0.166 |

PA: Physical activity; OBS: Oxidative Balance Score; Postmenopausal phases were categorized as follows: Post_0 (less than 1 year), Post_1 (1-2 years), Post_2 (2-3 years), and Post_3 (3-4 years) after menopause.

Supplementary Table 6. The accuracy and mean accuracy obtained through 5-fold cross-validation in the training set.

| Accuracy | TabPFN | Random Forest | XGBoost | CatBoost |
| --- | --- | --- | --- | --- |
| 1 | 0.816 | 0.816 | 0.802 | 0.831 |
| 2 | 0.816 | 0.821 | 0.797 | 0.816 |
| 3 | 0.754 | 0.763 | 0.754 | 0.763 |
| 4 | 0.820 | 0.791 | 0.743 | 0.811 |
| 5 | 0.733 | 0.762 | 0.782 | 0.772 |
| Mean accuracy | 0.788 | 0.800 | 0.782 | 0.808 |

TabPFN: Tabular Prior-data Fitted Network; XGBoost: Extreme Gradient Boosting; CatBoost: Categorical Boosting.

Supplementary Table 7. Evaluation metrics including ROC, F1 score, AUPRC, sensitivity, specificity, and accuracy for all four classification models in the testing set.

|  | TabPFN | Random Forest | XGBoost | CatBoost |
| --- | --- | --- | --- | --- |
| F1 score | 0.818 | 0.822 | 0.811 | 0.821 |
| Accuracy | 0.805 | 0.801 | 0.787 | 0.802 |
| Specificity | 0.826 | 0.802 | 0.777 | 0.816 |
| Sensitivity | 0.787 | 0.799 | 0.794 | 0.791 |
| AUPRC | 0.914 | 0.913 | 0.900 | 0.919 |

TabPFN: Tabular Prior-data Fitted Network; XGBoost: Extreme Gradient Boosting; CatBoost: Categorical Boosting; ROC: Receiver Operating Characteristic; AUPRC: Area Under the Precision-Recall Curve.

# Supplementary Figures


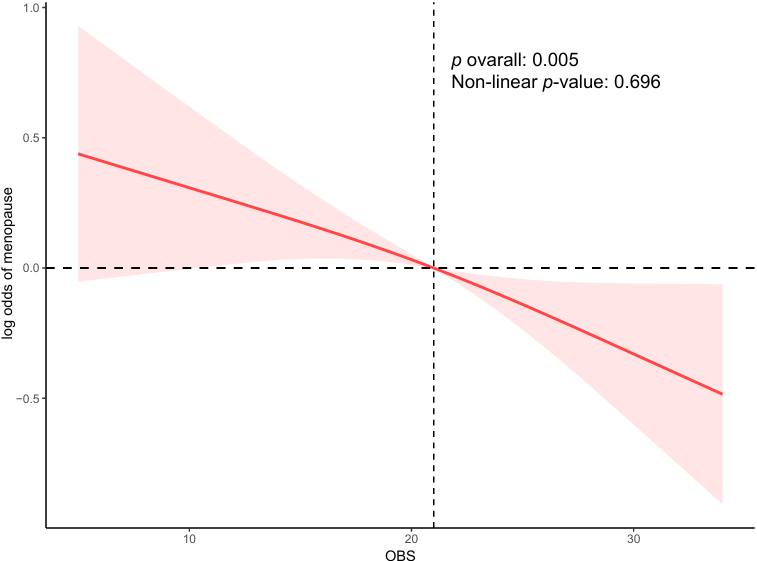


Suppelementary Figure 1. Association between OBS and the odds of menopause by Restricted Cubic Spline analysis. The selection of three knots of OBS yielding the minimum Akaike Information Criterion (AIC) value.


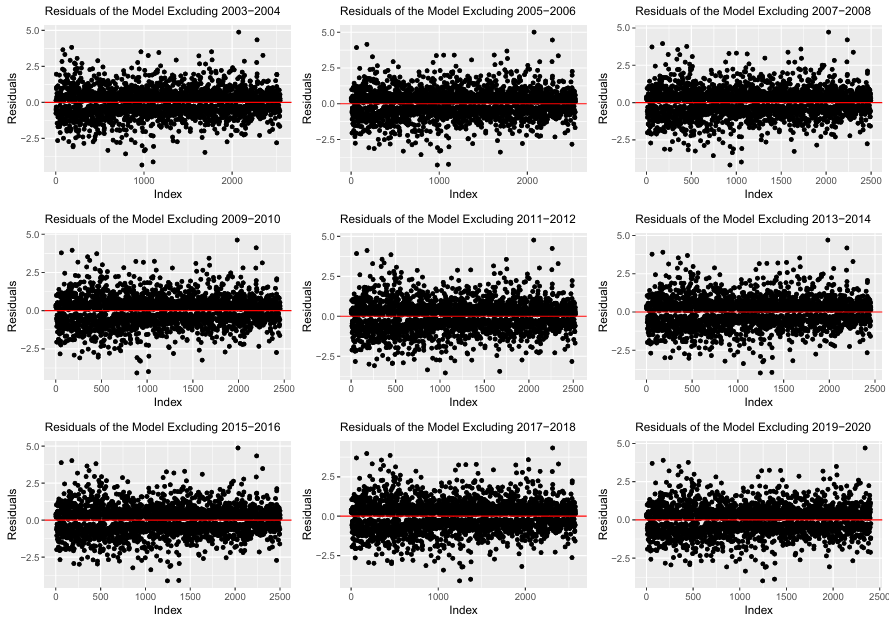


Supplementary Figure 2: Residuals of model performance excluding each survey years. The residual plots for Model 2, excluding individual survey years from 2003 to 2020. Each subplot displays the residuals against the index for the participants. The red horizontal line represents the zero residual line, indicating the ideal fit of the model. The distribution of residuals appears random and evenly spread around zero, suggesting that there is no evidence of overfitting in the model across the excluded survey periods.


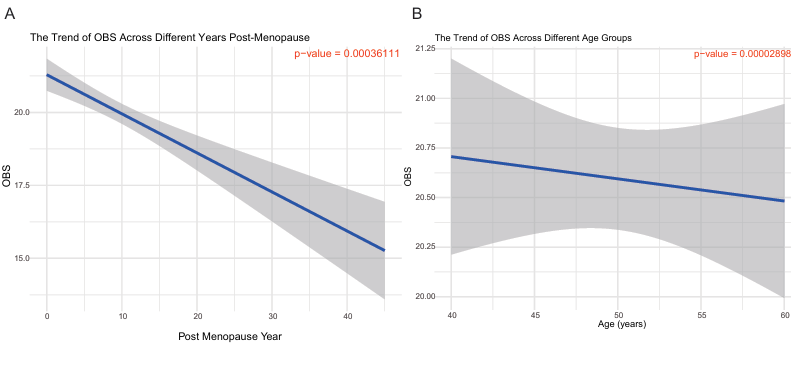


Supplementary Figure 3. Trends of OBS across different years post-menopause and age groups. Linear regression was employed after adjusting for confounding factors such as race, PIR, education, marital status, BMI, smoking, alcohol use, hypertension, CVD, DM, hyperlipidemia, daily energy intake, and fasting glucose levels. (A) The trend of OBS across various years post-menopause. (B) The trend of OBS across different age groups (40 to 60 years). OBS: Oxidative Balance Score; PIR: Poverty Income Ratio; BMI: Body Mass Index; CVD: Cardiovascular Disease;DM: Diabetes Mellitus;MET: Metabolic Equivalent of Task.
